# A Singular Case of Insanity

**Published:** 1858-07-01

**Authors:** 


### Art. VI.—A SINGULAR CASE OF INSANITY.

In an article entitled "Illustrations of Insanity, furnished by the Letters and Writings of the Insane," which was published in a previous number of the Journal,* Dr. Brigham introduced the following letter as a specimen of most entire incoherence :—

"My dear Sister—As the cedars of Lebanon have been walking through Edgeworth forest so long, you must have concluded that I have returned to the upper world, but I am still in purgatory for James K. Polk's sins, which, if they do not end in smoke, surely have as good a chance of beginning that way as the ideas began to shoot, for if T. had not left his trunk on the cart at the Depot, our shades would have been a deuced sight nearer to Land's End than Dr. Johnson said they would by the time the Yankees rebelled—(*ad interim*) —but I am now about between the porch and the altar, as Dr. —— used often to express himself, under the droppings of the sanctuary, where I wish to forget old things for a time at least, and return to some better place than the last. I could have kicked plagiary to the seventh seal.

"Do you know what this same long, taper roller is? well pop it off, if by their works ye shall know them. Pollock has as good a right to be a D.D. as that doctor we read of in Blackwood that sought so long for spoons and found them not, because they were all lead until they were new burnished in Holyrood palace, very near the place where Polk traced his pedigree, a little too near the loins of William the

* "American Journal of Insanity."

Conqueror, for the pleasures of memory or sense either, for Thompson, Bryant, Africaner, Ainsworth, or anybody else. I said I had been to the Poles, and S. had been there, and let T. Y. be witness that it was something more than stars, it is one thing neither you nor I can comprehend till we compare notes, but there is the least pit in hell that you ever saw, or ever will see, and a certain little white Devil just as ready now as ever to lend a helping hand to the cook to give her a lift over those bars. If you should ever be inclined to try Nebuchadnezzar's hollow furnace, for he did not wash all my guilt away did he? No indeed for he silvered my head nicely, so as to make it shine afar off. But the end of these things is not yet—consult S. I should like to see H. Honor to whom honor is due—tribute to whom tribute —give the Devil his due."

The writer, a student in one of the Eastern colleges, was brought to the institution about three months before the date of the above letter, labouring under an attack of acute mental disease, induced by too intense application to his studies. Six months afterwards he was discharged recovered; subsequently rejoined his class, and completed his college course with honour. He commenced the study of medicine, graduated at the College of Physicians and Surgeons in New York city, and connected himself with the New York Hospital, where he filled the office of house-physician with great credit to himself and advantage to the institution. Soon after the expiration of his term of office he had a fatal attack of remittent fever.

The following extract from his diary is furnished by his father. In connexion with the letter, it has a peculiar interest:—

"Yesterday I found in the 'American Journal of Insanity,' April, 1848 (p. 303), a copy of my letter written to Helen about six weeks before I left Utica. It is the most absurd medley of nonsense, but it recalled to my mind ideas of no little interest.

"My memory during the whole period of my violent illness was preternaturally active, calling up scenes and recollections of very early childhood; the toys and various utensils then about me, the little adventures and queer speeches which will cling to one's memory, while more important matters escape—these, and almost everything which, in a varied and not limited series of reading—names, scenes, historical and personal incidents, fact or fiction, phrases of other languages, passages of poetry and of the Bible —all these, by the merest similitude of sound, of name, or any other near or remote principle of association, were grouped in my mind, and would flit across its vision with inconceivable rapidity.

"Often, I remember, have I lain on my sleepless bed, and strung one group of words together, as they thus occurred to me, and, catching at some slight analogy in the last, would run off into another distinct series; and thus, till the tongue fairly wearied, and the lips refused to move, have arranged the affairs and settled the disputes of generations past, present, and yet to be; of princes and potentates, of injured queens, and defrauded heirs apparent—rummaging the legends of the Tower, and all the dark, romantic lore of Scottish feudal life; righting the wrong in every department or age of human existence, quarrelling most irreverently and pertly with many characters which good people deem sacred, and elevating in my own imagination many of those luckless but interesting heroes who, with many dazzling and redeeming qualities, had yet the misfortune to be wicked.

"Here came out in full my sneaking liking for Saul and Pontius Pilate (a very clever fellow, by the way, who occasionally appeared in the hall, and had an unfortunate squint), Henry VIII., Herod (whose valiant slaughter of Judea's infant-ry always inspired my young mind with a dread feeling of admiration), and Nebuchadnezzar. All these were living, breathing personages to me—for death seemed but a voluntary step, and a slight one—and with *these* I communed in the night-watches. I thought I heard them answer me, and I spoke as in reply—sometimes sadly, remembering some sorrowful scene gone by, with which I intimately connected them; sometimes in irrepressible glee; and again in anger—the mood varying with the turn of a word. Sometimes I would fall upon what, to me, was a sublime thought, and remembering Napoleon's saying, was pretty certain to change to a ludicrous interpretation, or some other such turn.

"Something of these fitful changes I recognise in this letter. It represents, tolerably well, the state of my mind—*very* well, for it is almost a transcript of what I would have said, if speaking to my sister. I well remember the day when the sheet of paper was brought me, upon which I wrote this, in a scrawl of a hand, for I dashed impetuously along, and what a sane person would say was an ill-spelled letter. But the spelling had its associations. This was the day, or one or two after, I had seen Helen.

"Shortly after I got one from *her* which most grievously distressed me. From it, I first realized that I was under restraint, and in an *Insane Asylum*. I held my head between my hands, and pressed it against the wall; every pulse came bounding with double force and rapidity; it seemed as if I *should* go mad then, and for ever. I did not notice those who passed—nor spoke, nor interested myself in the employments of others. I was *changing*.

"When Dr. Brigham passed through, I *begged* of him to take me from this place. I was too proud for that before. He tried to put me off. I followed him to the end of the hall, and then with my eyes till he passed out of sight.

"It was not many days before the doctor took me with him, as he went his rounds, and left me in a lower and a better hall. Then the scenes with which many of my delusions were connected, were changed. I looked no more at things around me through the distorted medium of an assumed character. I was H. B. M., not Mr. E. (my convenient $x$ character for any number of unknown personalities.)

"It was not without a voluntary effort, and *that* a painful one, that I tore myself from a glorious world of my own creating, and a throne of my own construction, to take my place in a real and very commonplace lower planet, full of ordinary and intractable characters. For did I not leave the inspiring and elevating society of the great, and good, and heroic of every age—and glorious schemes of empire—and grand ideas of improvement, whether commercial, or military, or literary, or in the fine arts? Were not tall monuments and noble temples to rise over this and every other land? Were not the thoughts of genius, expressed as they never before had found expression, to glow in fresco, on canvas, or to stand forth in pure dignity in the marble statue? Were not the scenes of my childhood's pleasures to be made sacred by its offering?

"Then should the pale scholar and the inspired poet no longer waste unheeded away, but each in his place should enjoy his fit reward. And the white sails of every nation, but rather of mine, should be spread to the breeze in every sea, bringing back richer freights than those of Solomon; and armies should stand ready at my bidding, innumerable, and comprising in their legions every force that ever in truth or poetry took the field—the battalions that contended with each other when there was war in heaven, the veterans of Napoleon, and the tiny squadrons of faëry land. But these I left: and, as I descended from my throne, reason resumed hers.

"Not many days afterwards, I wrote a most urgent letter home, as perfectly sane as ever I was or shall be, requesting to be removed.

"Day after day, and hours, and minutes I counted, till I reached my home—FREE."

